# The use of smartphones by junior doctors

**DOI:** 10.3402/meo.v20.28189

**Published:** 2015-06-02

**Authors:** Elliott Yann Ah-kee, Aamir Asif Khan

**Affiliations:** 1 Monklands Hospital, Monkscourt Ave, Airdrie, North Lanarkshire, ML6 0JS, UK; 2 Glasgow Royal Infirmary, 84 Castle Street, Glasgow, G4 0SF, UK

Smartphones have become omnipresent in our day-to-day living. The purpose-built software applications available on these phones have contributed to their emergence in clinical practice and increasing prevalence amongst junior doctors (American ‘Intern’ equivalent). The most commonly used apps include those for disease diagnosis/management, drug reference, clinical scoring systems, and medical calculators.

As junior doctors, we often use smartphone apps that provide hospital-specific guidelines and management algorithms for common conditions. This is extremely useful on busy wards with limited number of computers. Moreover, these apps provide rapid access to information in high-pressure situations such as being ‘on-call’. However, we are aware of the negative connotations associated with the use of smartphones in front of patients or senior colleagues. Their use is still considered unprofessional and often misinterpreted as texting, checking emails, or using social media. It is also sometimes perceived as laziness or as being uninterested by other members of hospital staff. A change in service user attitude towards smartphones may be the way forward in order to address these issues. Besides, new institutional policies to endorse the use of smartphones in clinical areas may also be required.

In a UK regional survey, Payne et al. (1) found that 78% (98/131) of junior doctors owned a smartphone and that 29.6% (29/98) used medical-related apps daily. The main finding of their survey was that junior doctors were keen to endorse such apps to enhance their clinical activities. A more recent similar survey conducted in the Republic of Ireland by O'Connor et al. (2) also showed high levels of smartphone ownership among junior doctors at 94.4% (102/108). However, of those, 80% made or received work-related texts and calls, 40% exchanged daily emails with senior trainees to provide updates on patients, and more than 50% had taken a work-related photo such as a wound for academic purposes or to obtain senior advice. In light of these findings, there is an ongoing debate as to whether the use of smartphones in a clinical setting leads to an erosion of core ethical principles. Further qualitative studies are required to investigate the perceptions of hospital staff on the use of smartphones in a clinical setting.

There is currently no governing body that regulates medical apps to ensure that their content is correct, up-to-date, and reliable. To address these issues, further in-depth focus on vigorous institutional policies is necessary to ensure that patient information is stored and transmitted securely on smartphones. Further hospital policies should also be explored to ensure that adequate guidance is in place for junior doctors with regards to the appropriate uses and restrictions of smartphones in clinical practice in order to preserve patient confidentiality and safety.

We note with interest that a smartphone app recently developed for hospital staff in the emergency department at the Royal and Broadgreen hospitals, UK, is currently being used by junior doctors to check up-to-date guidelines on common acute conditions at the patient's bedside (3). In addition, the app also contains links to helpful contacts and Trust policies, and has been downloaded by health care professionals in over 50 countries. The positive feedback received has led to the app becoming an integral part of the hospital training protocol. Similar initiatives may be the way forward in the future for a more efficient training and education of new medical graduates. In conclusion, it is undeniable that the use of personal smartphones in clinical practice is an established reality, particularly amongst junior doctors. Smartphone apps provide user-friendly and intuitive interfaces for junior doctors to efficiently perform their daily clinical tasks.
